# Editorial: Cancer Immunotherapies: From Efficacy to Resistance Mechanisms

**DOI:** 10.3389/fimmu.2022.939789

**Published:** 2022-06-02

**Authors:** Janin Chandra, Morten Hansen, Nathalie Labarriere, Ilaria Marigo, Fernando Souza-Fonseca-Guimaraes, Lazar Vujanovic, Yoshinobu Koguchi, Nicolas Jacquelot

**Affiliations:** ^1^ The University of Queensland Diamantina Institute, Faculty of Medicine, The University of Queensland, Woolloongabba, QLD, Australia; ^2^ National Center for Cancer Immune Therapy (CCIT-DK), Department of Oncology, Copenhagen University Hospital, Herlev, Denmark; ^3^ Nantes Université, Université Angers, INSERM, Immunology and New Concepts in ImmunoTherapy, INCIT, UMR 1302., Nantes, France; ^4^ LabEx IGO, Université de Nantes, Nantes, France; ^5^ Veneto Institute of Oncology IOV-IRCCS, Padova, Italy; ^6^ Department of Surgery, Oncology and Gastroenterology, University of Padova, Padova, Italy; ^7^ UPMC Hillman Cancer Center, University of Pittsburgh, Pittsburgh, PA, United States; ^8^ Department of Otolaryngology, University of Pittsburgh, Pittsburgh, PA, United States; ^9^ Earle A Chiles Research Institute, Providence Cancer Institute, Portland, OR, United States; ^10^ Princess Margaret Cancer Centre, University Health Network, Toronto, ON, Canada

**Keywords:** cancer, immunotherapy, tumor microenvironment, immune checkpoint blockers, adoptive T cell therapy,

Current generations of cancer immunotherapies are used to abrogate immunosuppression or modify immune cells to enhance anti-tumor immune cell functions. With recent advances in antibody generation, characterization and production, the development of monoclonal antibody-based immunotherapies targeting co-inhibitory receptors on T cells, the so-called “immune checkpoint blockade (ICB)”, has fundamentally changed the outlook on metastatic cancer and has offered new hope to patients for long-term survival. Despite their success, the clinical efficiencies of these treatments remain low and vary across different malignant diseases, sparking effort from the community to identify mechanisms responsible for therapy resistance and to offer a rationale for combination therapies aiming to further improve patient prognosis and outcomes. Under the theme “Cancer Immunotherapies: from efficacy to resistance mechanisms”, 41 articles covering a wide range of topics in cancer immunotherapy were contributed. Here, we highlight the most valuable insights from original research articles and reviews published in this issue.

## Adoptive T Cell Therapy

Adoptive T cell therapy (ACT) utilizes patient’s own immune cells to seek out and kill tumor cells. Kumar et al., reviewed the latest developments and highlighted future perspectives of ACT in cancer. One novel approach developed by Cheng et al., consists of injecting expanded autologous circulating cells that were sequentially primed with dendritic cells loaded with 6B11 minibody, mimicking the ovarian cancer–associated antigen OC166-9, and expanded with IL-2 and immobilized anti-CD3 antibody. This preliminary study demonstrated a good safety profile and showed potential efficacy against platinum-resistant recurrent or refractory ovarian cancer (Cheng et al.), warranting further evaluation. Other approaches involve the expression of chimeric antigen receptors (CAR) targeting tumor-associated antigens in T cells. The infusion of CAR T cells has shown great efficacy against haematological malignancies. However, this therapy is often associated with inflammatory toxicities requiring therapeutic intervention. In this regard, Fischer and Bhattarai summarized our current understanding of mechanisms contributing to adverse inflammatory responses, described current strategies for the management of toxicities, and reviewed ongoing developments to prevent harmful inflammatory toxicities associated with CAR T cell treatment (Fischer and Bhattarai).

## Impact of the Tumor Microenvironment

The tumor microenvironment (TME) refers to the complex local tumor ecosystem composed of tumor cells, extracellular matrix, vascular and lymphatic vessels, fibroblasts, and immune cells. Each component of the TME influences the efficacy of current immunotherapy treatments. Recent evidence suggests that not only T lymphocytes but also innate immune cells drive ICB efficacy and may represent novel immunotherapy targets. Shaver et al., reviewed the role of natural killer (NK) cells in ICB efficiency and described current advances in treatments that harness NK cell function. Patients who respond favourably to ICB often have high tumor infiltration by T cells or NK cells. However, this is not a promise of success as multiple primary and acquired resistance mechanisms to immunotherapy have recently been described.

## Primary and Acquired Resistance Mechanisms

Despite their unprecedented success, most patients do not respond favourably to ICB therapy (primary resistance) or initially respond to but subsequently acquire resistance to treatments and relapse. In this issue, Wang et al., provided an exhaustive summary of currently identified primary and acquired resistance mechanisms. Complementary to this work, Zhou et al., reviewed underlying mechanisms and potential strategies to overcome acquired resistance mechanisms to ICB, notably through combination treatments. Many identified resistance mechanisms relate to specific TME composition and innate or adaptive immune cell dysfunction. Li et al., comprehensively reviewed the role of inflammasomes within the TME, which play a key role in regulating the state of inflammation and recruitment of immune cells to the tumor (Li et al.). They describe non-canonical pathways of inflammasome activation that lead to expulsion of ‘nets’ of DNA from neutrophils (NETosis), which promote tumorigenesis by shielding cancer cells from immune cell attacks (Li et al.). These DNA ‘nets’ further promote tumor cell mobility and thereby enhance metastasis. Cai et al., highlighted how anti PD-(L)1 targeting agents negatively impact macrophage reprogramming leading to reduced efficacy of the treatment. Chen et al., demonstrated that the CD47:SIRPa signalling pathway contributed to reduce phagocytic responses of macrophages, negatively impacting antibody-based treatments in chronic lymphocytic leukaemia patients. In colorectal cancer, patients harboured increased TIGIT expression on their circulating CD3^+^ T cells compared to healthy donors (Shao et al.). Shao et al., further showed that TIGIT expression was associated with T cell dysfunction and impaired metabolism, negatively impacting patient prognosis.

Brain cancers are particularly resistant to immunotherapies. Yu and Quail extensively discussed how intra- and inter-tumoral heterogeneity, immunosuppressive TME and tumor plasticity, together with unique cancer location within the central nervous system are all challenges for immunotherapy of glioblastoma. To address these challenges, many drug candidates for glioblastoma are currently developed and tested as standalone treatments or in combination with current ICB (Yu and Quail). In neuroblastoma, Tang et al., found that high levels of *CD4* and *NKG2C/E* gene expression were both associated with prolonged patient survival, suggesting that CD4^+^ cytotoxic cells could provide protective anti-tumor immunity and might represent an attractive target for future drug development.

## Overcoming Resistance Mechanisms Using Combination Treatments

Several articles in this issue discussed novel combinatorial approaches to improve responses to ICB. These include anti-TIGIT (Shao et al.) or GARP : TGF-β1 (Bertrand et al.) antibodies, inhibitors of indoleamine 2,3-dioxygenase 1 (IDO1) (Li et al.), Wnt/b-catenin (Huang et al.), proteasome (Renrick et al.), or icaritin (Tao et al.), a prenylflavonoid derivative. Using the MC38 adenocarcinoma cell line, Bertrand et al., described increased anti-tumor responses when anti-PD-1 antibody was combined with GARP : TGF-β1 antibodies, that led to improved outcomes resulting from blocking both active TGF-β1 production and the latent form covalently bound to GARP expressed by regulatory T cells. Anti-tumor responses were associated with increased T cell infiltration and density of blood vessels (Bertrand et al.). In triple-negative breast cancer (TBNC) patients, IDO1 and PD-L1 are both highly expressed in the TME, constituting a main obstacle to anti-tumor immune responses. Based on these observations, Li et al., found that dysfunctional γδT cells had restored cytotoxic and anti-tumor functions against breast cancer cells upon treatment with an IDO1 inhibitor, but not with an anti-PD-L1 antibody, *in vitro* and *in vivo*, suggesting a rationale for using IDO1 inhibitors to treat cancer patients. Since a phase III clinical trial in melanoma testing IDO1 inhibitors in combination with anti-PD-1 antibody did not improve patient survival (1), targeting IDO1 may require more sophisticated approaches to enhance the effectiveness of anti-cancer treatments. Although much less studied, Mondanelli et al., reviewed existing evidence and current challenges for IDO2, the IDO1 paralog, as a target in cancer immunotherapy.

Other inhibitors have also been proposed. Huang et al., described the capacity of an inhibitor of the Wnt/b-catenin pathway to synergize with anti-PD-L1 antibody to improve intratumoral CD8 effector function. Renrick et al., showed that the proteasome inhibitor bortezomib, a drug approved for multiple myeloma and mantle cell lymphoma that sensitizes tumor cells to apoptosis, can increase anti-tumor T cell function by increasing the expression of miR-155 that subsequently lowers PD-1-mediated T cell exhaustion and enhances cytotoxic effector functions in animal models of solid tumors. Beyond the use of antagonistic antibodies and inhibitors, the exploration of natural components in cancer therapy is also evolving. Tao et al., showed that the prenylflavonoid derivative icaritin suppresses tumor growth by blocking myeloid derived suppressor cells in murine models of hepatocellular carcinoma, and enhances anti-tumor efficacy of PD1-targeting (Tao et al.).

Several case reports suggested the combination of ICB with endocrine therapy, radiotherapy, or chemotherapy. Wu et al. described two cases of hormone receptor positive metastatic breast cancer that responded to endocrine therapy in combination with PD-1 ICB, where metastases were located in either brain or bone, both anatomically challenging sites and not easily accessible for surgical removal (Wu et al.). Wu et al., reported a case report of a metastatic renal cell carcinoma (RCC) patient, who was resistant to ICB and radiotherapy, but started displaying abscopal effects and ICB response of metastases after removal of the primary tumor mass (Wu et al.). At this stage, it is unclear how cytoreductive surgery leads to abscopal effects, a well-known phenomenon in RCC, and the authors speculate that the total amount of PD-L1 expressing tumor cells was too large for ICB therapy to be effective, and radiation induced memory T cells could only become effective after the global tumor cell burden was significantly reduced by removing the largest tumor mass on the kidney. Xiu et al., described two cases of lung adenocarcinoma with epidermal growth factor receptor mutations and resistant to tyrosine kinase inhibition, where one was treated with a combination of chemotherapy, and the other received chemotherapy and PD-1 ICB (Xiu et al.). In this instance, the patient receiving the chemo/ICB combination acquired a partial response while the patient receiving chemotherapy only progressed.

Chen et al., provided an interesting perspective on the use of IL-10 as a cancer immune therapeutic. While IL-10 is largely associated with immune suppression, this original research study demonstrates that intra-tumoral delivery of IL-10 leads to significantly reduced tumor growth and, in combination with an oncolytic adenovirus, profoundly limits tumor growth in a CD8^+^ T cell-dependent manner (Chen et al.).

Extracellular vesicles secreted by cancer cells also contribute to treatment resistance through transmission of drug transporters that are taken up by target cells, which induces drug resistance and confers survival advantage. Hekmatirad et al., showed that inhibition of exosome release increases the susceptibility of acute myeloblastic leukemic cells to PEGylated liposomal doxorubicin, a combination warranting further evaluation using *in vivo* models.

## Immunotherapy for Bladder Cancer

Immunotherapies are commonly used to treat bladder cancer patients. The ICB response rate for advanced bladder cancer, like many other solid cancers, lies around 20%. Therefore, identification of predictors of response and alternative therapy regimens are needed. Lin et al., reported that mutations in the nuclear receptor corepressor 1 (NCOR1) are significantly associated with response and overall survival. NCOR1 mutation was further associated with neoantigen load and TIL infiltration (Lin et al.). Bacillus Calmette-Guerin (BCG) treatment, a live-attenuated *Mycobacterium bovis* vaccine traditionally used to prevent tuberculosis, is a standard immunotherapy for non-muscle invasive bladder cancer delivered through intravesical instillation. Lim et al., provided an in-depth characterization of the changes in immune composition in responders of BCG therapy, demonstrating that an infiltration of PD-1^+^ effector CD8^+^ and non-Treg CD4^+^ T cells is indicative of response and better recurrence-free survival (Lim et al.). Dowell et al., observed the constitutive expression of PD-L2 in both bladder cancers and in normal urothelium, and discussed its potential role in maintaining tolerance of this immune-privileged anatomical site (Dowell et al.). Finally, in a case report, Cao et al., described a patient with muscle invasive bladder cancer who achieved bladder preservation through a combination of neoadjuvant chemotherapy with anti-PD-1 antibody (Cao et al.). While these first case reports of experimental combination approaches are promising, appropriately controlled clinical trials are only slowly emerging.

## Prognostic/Predictive Biomarkers for Patient Outcomes

Development of biomarkers for patient outcomes is critical for patient stratification and deployment of effective cancer immunotherapy. Patient’s age, tumor mutation, and gene polymorphism were shown to be associated with different response rates to ICB treatment in melanoma. Safi et al., demonstrated that the age of the patients receiving ICB influenced clinical outcomes with younger patients having improved survival compared to older groups. Parakh et al., found an association between germline PD-1 polymorphism and progression free-survival (PFS) in response to anti-PD-1 antibody treatment. In an Asian cohort, Zhou et al., uncovered that patients with NRAS mutated tumors have lower response rates to anti-PD-1 antibody treatment associated with reduced survivals. Additional host- and tumor-intrinsic characteristics were described and correlated with clinical prognosis, some having the potential to predict ICB efficiency, providing rationale for more individualized clinical management strategies (2). Using the TCGA datasets, Fan et al., described a gene signature composed of five interleukins and/or receptors associated with better prognosis in lung adenocarcinoma. Complementary to this work, Munari et al., found that a high CD8^+^ T cell density in the TME was associated with improved survival in non-small cell lung cancer. Retrospective analyses of the TME transcriptomic profile from melanoma and urothelial cancer patients have revealed that high proportions of activated T cells, M1 macrophages and follicular T helper cells were associated with improved clinical outcomes following ICB, while mast cells or resting memory CD4^+^ T cells correlated with dismal prognosis (Liu et al.). In addition to these tumor-intrinsic parameters, Billon et al., found that high baseline plasmatic levels of BTN2A1, a molecule binding to γδTCR and activating γδT cells, was significantly associated with shorter PFS of metastatic RCC patients treated with anti-PD-1 antibody, suggesting a potential role of γδT cells in ICB efficacy. A network meta-analysis performed by Botticelli et al., in head and neck cancer deciphered potential association between the efficacy of anti-PD-1 and anti-PD-L1-based treatment with clinical covariates. The authors suggested that anti-PD-1 therapy seems to be more efficient in smoking patients and human papilloma (HPV) negative cases while anti-PD-L1 antibody might be more effective in female patients, locally recurrent settings and in HPV positive cases. In advanced gastric cancer patients, sarcopenia, characterized by reduced skeletal muscle mass, combined with a high neutrophil to lymphocyte ratio was associated with reduced efficacy of anti-PD-1 antibodies (Kim et al.). Collectively, these studies provided key insights into the importance of the tumor immune contexture and clinical covariates in dictating clinical prognosis and treatment efficacy in cancer patients, warranting validation for patient stratification and selection in clinical practice.

## Future Perspectives

Aforementioned reviews and original articles critically highlight the importance of studying cancer biology *via* global approaches. The use of systems biology approaches to comprehensively understand primary and acquired resistance mechanisms to ICB at play should pave the way to more personalized medicine, taking into account an individual’s genetic and environmental influences such as COVID-19 (El-Shakankery et al.). However, despite intensive research efforts, many of the identified mechanisms are still poorly understood. In this regards, Sun et al. provided a comprehensive review of the DNA damage repair pathways in tumors and their relation to ICB response. Mismatch repair is a recognized predictive biomarker for ICB, and original research suggests a combination of agents targeting DNA damage repair and ICB and hold promises for increasing therapeutic efficacy. However, this field of research is complex as different pathways will lead to different outcomes, where for example high mutational load is associated with increased ICB response while increased somatic copy numbers are associated with decreased outcomes and immune suppression likely due to the accumulation of unfavourable mutations. This example illustrates the duality of the problem with pathways playing often opposite roles according to the cell type or tissue involved and the lack of sensitive tools and methods to accurately predict patient responses to ICB.

In a near future, with the development and use of artificial intelligence and machine learning algorithms, we anticipate the development of accurate nomograms integrating multiple variables for patient stratification and treatment decision. With this regard, Yuan et al., developed and validated a nomogram using pre-treatment contrast-enhanced computed tomography (CT) images and clinical risk factors to estimate the anti-PD-1 treatment efficacy in hepatocellular carcinoma patients. The developed model showed high specificity and sensitivity, warranting further validation in a prospective setting. In addition, with the advancement of technologies providing high-dimensional data, we are slowly uncovering the complexity of the highly heterogenous landscapes of malignant diseases. In head and neck squamous cell carcinoma patients, Kulasinghe et al. used highly multiplexed digital spatial profiling to unravel immune cell types and markers correlating with progressive disease, revealing potential biomarkers and their spatial distribution *in situ*. The application of spatial high-dimensional transcriptomic and proteomic technologies with single cell resolution represents an essential field of development to guide biomarker and target discovery and the foundation for personalized cancer therapy.

In summary, a better understanding of resistance mechanisms will allow clinicians and scientists to design novel targeting approaches or to optimally orchestrate combinatory treatments aiming to overcome resistance mechanisms with the goal to improve clinical outcomes in a more personalized manner.

## Author Contributions

NJ and JC wrote the initial draft. All authors listed, have made substantial, direct, and intellectual contribution to the work, and approved it for publication.

## Funding

This work was supported by grants from Cure Cancer Australia and Cancer Australia through the Cancer Australia Priority-driven Cancer Research Scheme (APP1163990 to NJ, 1158085 to FS-F-G), Cancer Council NSW (RG21-05 to NJ), the US Department of Defense – Breast Cancer Research Program – Breakthrough Award Level 1 (#BC200025 to FSFG), a ANZSA Sarcoma Research Grant (supported by Kicking Goals for Xav, Stoney’s Steps and Stop Sarcoma to FS-F-G), National Institute of Dental & Craniofacial Research (LV; 1R01DE031947-01), Euronanomed 2019-135 and 2021-014 (IM) and a UQ Diamantina Institute Laboratory Start-Up Package (to FS-F-G).

## Conflict of Interest

YK receives funding from Tesaro, a GSK company, Bristol-Myers Squibb, and Shimadzu Corporation. LV is a co-inventor of a methodology licensed to INmune Bio, Inc. where a selective inhibitor of soluble TNF can be used to prevent or treat malignancies. MH receives funding from ImmuniTrack A/S as part of a consultancy agreement.

The remaining authors declare that the research was conducted in the absence of any commercial or financial relationships that could be construed as a potential conflict of interest.

## Publisher’s Note

All claims expressed in this article are solely those of the authors and do not necessarily represent those of their affiliated organizations, or those of the publisher, the editors and the reviewers. Any product that may be evaluated in this article, or claim that may be made by its manufacturer, is not guaranteed or endorsed by the publisher.
